# Supplementary material for: Association of faecal pH with childhood stunting: Results from a cross-sectional study
Source: BMJ Paediatr Open. 2019 Sep 13;3(1):e000549. doi: 10.1136/bmjpo-2019-000549 (PMC6782033; doi:10.1136/bmjpo-2019-000549)
Supplement: Author's manuscript [file bmjpo-2019-000549.draft_revisions.pdf]

BMJ Paediatrics Open

BMJ Paediatrics Open is committed to open peer review. As part of this commitment we make the peer review history of every article we publish publicly available.

When an article is published we post the peer reviewers' comments and the authors' responses online. We also post the versions of the paper that were used during peer review. These are the versions that the peer review comments apply to.

The versions of the paper that follow are the versions that were submitted during the peer review process. They are not the versions of record or the final published versions. They should not be cited or distributed as the published version of this manuscript.

BMJ Paediatrics Open is an open access journal and the full, final, typeset and author-corrected version of record of the manuscript is available on our site with no access controls, subscription charges or pay-per-view fees (http://bmjpaedsopen.bmj.com).

If you have any questions on BMJ Paediatrics Open's open peer review process please email info.bmjpo@bmj.com

BMJ Paediatrics Open

Fecal pH: Is it associated with linear growth of children?

Journal:	*BMJ Paediatrics Open*
Manuscript ID	bmjpo-2019-000549
Article Type:	Original article
Date Submitted by the Author:	30-Jun-2019
Complete List of Authors:	Hossain, Md. Shabab; International Centre for Diarrhoeal Disease Research Bangladesh,<br>Das, Subhasish; International Centre for Diarrhoeal Disease Research Bangladesh<br>Gazi, Md. Amran; International Centre for Diarrhoeal Disease Research Bangladesh<br>Alam, Md. Ashraful ; International Centre for Diarrhoeal Disease Research Bangladesh<br>Haque, Nur Muhammad Shahedul ; International Centre for Diarrhoeal Disease Research Bangladesh<br>Mahfuz, Mustafa; International Centre for Diarrhoeal Disease Research Bangladesh<br>Ahmed, Tahmeed; International Centre for Diarrhoeal Disease Research Bangladesh, Nutrition and Clinical Services Division (NCSD)<br>Damman, Chris J. ; University of Washington; Bill and Melinda Gates Foundation
Keywords:	Growth

SCHOLARONE™
Manuscripts

Fecal pH: Is it associated with linear growth of children?

Md. Shabab Hossain[1], Subhasish Das[1], Md. Amran Gazi[1], Md. Ashraful Alam[1], Nur Muhammad Shahedul Haque[1], Mustafa Mahfuz[1], Tahmeed Ahmed[1], Chris J. Damman[2,3]

[1]Nutrition and Clinical Services Division, International Centre for Diarrheal Disease Research, Bangladesh (icddr,b), Dhaka, Bangladesh

[2]Program Officer, Bill and Melinda Gates Foundation, Seattle, Washington, USA

[3]Division of Gastroenterology, Department of Medicine, University of Washington, Seattle, Washington, USA

Name and email of all authors:

Md. Shabab Hossain: dr.shabab@icddrb.org

Subhasish Das: subhasish.das@icddrb.org

Md. Amran Gazi: amran.gazi@icddrb.org

Md. Ashraful Alam: mashraful@icddrb.org

Nur Muhammad Shahedul Haque: shahedhaq@icddrb.org

Mustafa Mahfuz: mustafa@icddrb.org

Tahmeed Ahmed: tahmeed@icddrb.org

Chris J. Damman: Chris.Damman@gatesfoundation.org

Address for correspondence:

Tahmeed Ahmed

Nutrition and Clinical Services Division, International Centre for Diarrhoeal Disease Research, Bangladesh (icddr,b), 68 Shaheed Tajuddin Ahmed Sarani, Mahakhali, Dhaka, Bangladesh

Phone: +880 1713044799; Email: tahmeed@icddrb.org

Confidential: For Review Only

Key words: Fecal pH; growth; children; Bangladesh

Manuscript Word count: 2485

Number of figures: 01

Number of tables: 02

Source of Funding

This protocol is supported by the Bill and Melinda Gates Foundation under its Global Health Program. Project investment ID is OPP1136751.

(https://www.gatesfoundation.org/How-We-Work/Quick-Links/Grants-Database/Grants/2015/11/OPP1136751)

Trial registration number

ClinicalTrials.gov ID: NCT02812615

(https://clinicaltrials.gov/ct2/results?cond=NCT02812615&term=&cntry=&state=&city=&dist=)

Conflicts of Interest

None declared

Acknowledgements

This research protocol was funded by *the Bill and Melinda Gates Foundation (BMGF) under its Global Health Program. Project investment ID is OPP1136751*. icddr,b acknowledges with gratitude the commitment of the BMGF to its research efforts. icddr,b is also grateful to the Governments of Bangladesh, Canada, Sweden and the UK for providing core/unrestricted support. We express our sincere thanks to our colleagues at the International Centre for Diarrhoeal Disease Research, Bangladesh and the study participants.

Confidential: For Review Only

Abstract

Background: Gut microbiota plays an important role in growth of children. The gut of children with optimum linear growth is enriched in certain species, especially *Clostridia* and *Bifidobacteria*. *Bifidobacteria* and commensal *Clostridia* both contribute to formation of acidic stool, and an elevated fecal pH indicates reduction of these species in the gut. The purpose of the study was to investigate the association of fecal pH with linear growth in children.

Methods: One hundred children with length-for-age Z score (LAZ) <-1 aged between 12-18 months were enrolled from the ongoing Bangladesh Environmental Enteric Dysfunction (BEED) study conducted in Dhaka, Bangladesh. LAZ was measured by anthropometry and data on factors affecting linear growth was recorded. Fecal pH measurement was done using pH meter on freshly collected non-diarrheal fecal samples following standard procedure. Multivariate linear regression was done to quantify the relation between fecal pH and LAZ scores.

Results: The mean LAZ and fecal pH of the children were -2.12±0.80 and 5.84±1.11 respectively. Pearson correlation analysis showed a statistically significant negative correlation between stool pH and the LAZ scores ($p<0.05$). After inclusion of other factors affecting linear growth into the regression model, a statistically significant positive association was observed between maternal height with LAZ score while a significantly inverse association was observed between fecal pH and LAZ score ($p<0.05$).

Conclusion: Maternal height and fecal pH had significant association with linear growth. While previous studies have already established the former one, fecal pH has emerged as a possible determinant of linear growth of children.

Confidential: For Review Only

Introduction

Malnutrition accounts for almost half of all deaths in under five children, estimated to be more than 3 million child deaths annually (1). The effect is more pronounced in children living in low-and-middle-income countries (LMICs) (1). Chronic malnutrition, which is manifested as linear growth failure or stunting (2), is the most common form of malnutrition across the globe, affecting about one-third of children in developing countries (3). Among them, southern Asia harbors the maximum number of stunted children (4). Stunted children suffer from reduced neuro-developmental potential and poor cognitive function (2), thus fail to reach their full developmental potential (5), leading to sub-standard educational performance and economic productivity in the later phases of life (6). The etiology of stunting is poorly understood (4) and though poverty-associated food insecurity might play a vital role, the etiology of this condition is far more complex (7). Several factors including intrauterine growth retardation, mother's height, impaired immunity and recurrent infections, environmental enteric dysfunction (EED), neuro-endocrine and hormonal factors, and host genetic factors compounded by food insecurity are all part of this vicious cycle (8-13). Recent studies suggest that alteration in the gut microbiota is also a part of this cycle (4, 7-9, 14).

A healthy gut microbiota is essential to human health (7). It performs diverse protective, structural, and metabolic functions of the gut; for instance, production of short-chain fatty acids (SCFA), which act as the primary nutrient source for the colonic epithelium, intestinal epithelial cell proliferation and development of the mucosal immune system, production of certain vitamins, promoting mineral absorption, influencing metabolic rates and appetite, induction of host genes for nutrient uptake; all of which are critical for optimal nutrient absorption (8, 15-17). On the other hand, dysbiosis or an altered microbiota composition, which refers to reduction or absence of a species or presence of an inflammogenic species could lead to a number of disease states or malnutrition even in the face of adequate food intake (7).

Confidential: For Review Only

Despite the fact that chronic malnutrition is more prevalent than acute malnutrition globally, most published studies have concentrated on the role of gut microbiota in acute malnutrition. The limited studies that exist show that, gut microbiota of stunted children are predominant in bacteria belonging to the inflammogenic taxa including those in the Desulfovibrio genus and Campylobacterales order (4). On the contrary, the gut microbiota of children with optimum linear growth are enriched in certain species, especially the commensal *Clostridia* (18) and *Bifidobacteria* (4, 7), which is referred to as the keystone infant gut symbiont (19). Fecal pH, a probable determinant whose relationship with linear growth has never been explored to the best of our knowledge, is strongly driven by the abundance of the butyrate producing commensal *Clostridia*, especially clusters XIVa and IV, and *Bifidobacteria* in the gut (19, 20). These commensals lower the pH in the colon contributing to formation of a more acidic stool and the reduction or loss of these species lead to an elevation in fecal pH (19).

Therefore, elevated fecal pH indicates a marked change in the infant gut microbiome, specifically a profound reduction of the mentioned species. Since fecal pH is an indirect yet novel tool for assessing commensal *Clostridia* and *Bifidobacteria* abundance in the infant gut, while their abundance share a significant association with linear growth, we made an attempt to determine the association between linear growth and fecal pH and to investigate if fecal pH is associated with linear growth in early childhood.

Methods

Study site and Data collection

This study was a part of the ongoing Bangladesh Environmental Enteric Dysfunction (BEED) study which is being conducted among the residents of *Bauniabadh* slum in Mirpur, Dhaka. BEED study is a community-based nutrition intervention study aimed to validate non-invasive biomarkers of environmental enteric dysfunction (EED) and better understand the disease pathogenesis. The details and

Confidential: For Review Only

overall design of the BEED study have already been published elsewhere (21). A written informed consent was obtained from the parents of children after explaining the aims and procedures of the study. For this particular study, a total of 100 children aged between 12-18 months with length-for-age Z-scores (LAZ) <-1 were enrolled from the same community from September 2018 to February 2019. Weight and length of the child along with mother's height was measured using standard protocol. Data regarding socio-economic, morbidity, dietary intake and breastfeeding was collected using hard copy questionnaires at the time of enrolment and prior to sample collection. Dietary intake was assessed using qualitative food frequency questionnaires (FFQ) in which individual food items that was fed to each child during the last 24 hours were recorded. This research was done without patient involvement. Patients were not invited to comment on the study design and were not consulted to develop patient relevant outcomes or interpret the results. Patients were not invited to contribute to the writing or editing of this document for readability or accuracy. Ethical approval was taken from Institutional review committee of icddr,b.

Measuring fecal pH

Following standard operating procedures (19, 22), fresh non-diarrheal fecal samples were collected immediately after defecation and 1-2 grams of stool sample was kept in a sterile specimen container. 10 ml of de-ionized water was mixed and homogenized with this stool properly. The probes of the pH meter were completely dipped into this mixture and kept for one minute and the reading was recorded. The procedure was done within the first 2 hours of sample collection. The test was carried out using portable stool pH meter (Hanna Instruments, USA).

Small intestinal bacterial overgrowth (SIBO)

Presence of small intestinal bacterial overgrowth (SIBO) was determined by hydrogen breath test from breath samples taken from malnourished adults using BreathTracker SC (QuinTron Instrument Company, Milwaukee, USA). A total of ten breath samples were collected per case including a 3-hr fasting baseline sample followed by nine more samples at 20 minutes intervals after ingestion of glucose at 1 gram/kg

Confidential: For Review Only

body weight diluted in 5 ml/kg of water. A single difference of ≥ 12 ppm of hydrogen value from fasting baseline was considered to be SIBO positive (23).

Statistical analyses

Statistical analyses were performed using SPSS version 20.0 (IBM). Mean values and standard deviation (SD) of means were used to describe the distribution. Correlation between stool pH and LAZ scores was done by bivariate correlation analysis using Pearson correlation and scatter plots. As LAZ is the outcome variable, at first bivariate linear regression was performed between LAZ scores with each individual factor. Results with a significance level below 0.2 were included in the multivariate regression model. In addition, age and sex were included in the final model due to the inconsistent growth pattern in children of either sex in this age group (24) and minimum dietary diversity (MDD) was included in the final model instead of the individual food groups. Multivariate linear regression was done to quantify the relation between fecal pH and LAZ scores after adjusting for age, sex, maternal height, MDD, breastfeeding, morbidity, antibiotics taken in the last 14 days, SIBO and incorporation of several aspects of socio-economic status: access to improved **W**ater and sanitation, the selected approach to measuring household wealth (**A**ssets), **M**aternal education, and **I**ncome (i.e. the WAMI index) (25).

Results

A total of 100 children with mean age of 15±2 months underwent fecal pH examination. Among them, 57 (57%) were female and 43 (43%) were male. Table 1 shows the socio-demographic characteristics of the study population.

The mean LAZ and fecal pH of the children were -2.12±0.80 and 5.84±1.11 respectively. Pearson correlation between fecal pH and LAZ scores showed a statistically significant negative correlation (-0.46, p=0.00). Scatter plot (Figure-1) showed a similar correlation between stool pH and the LAZ scores as well.

Confidential: For Review Only

Results of the bivariate linear regression with a significance level below 0.2 and in addition age, sex and MDD were included in the multivariate regression model. A statistically significant inverse association was observed between fecal pH with LAZ score while a significantly positive association was observed between maternal height and LAZ score of the children (Table 2). A single unit increase in fecal pH resulted in 0.34 unit decrease in the LAZ score, while a single unit increase in maternal height caused 0.04 unit increase in the LAZ of the child.

Discussion

Our study revealed that, fecal pH has a significant association with linear growth in early childhood. To our knowledge, this was the first study to explore the relationship between fecal pH and linear growth in children. Though studies have shown significant association of fecal pH with severe acute malnutrition (SAM) (26), a thorough literature review yielded no results on the relationship between fecal pH with linear growth.

A trend of increasing infant fecal pH in children is observed over the past century which demonstrated a change in fecal pH from 5.0 to 6.5 from 1926 to 2017 (19). This elevation of fecal pH is consistent with reported reduction of certain important gut microflora. Factors responsible for reduction of these important microbiota from the infant gut over the past century comprises of an increase in consumption of human milk replacers (e.g., infant formula) which lack the bacterial selectivity of human milk (19), increased caesarean section delivery which limits the natural fecal-oral transfer from mother to infant associated with vaginal delivery (27) and overuse of antibiotics which alter the acquisition of gut microbes by the infant (28). Butyrate producing commensal *Clostridia* and *Bifidobacteria* both contribute to formation of acidic stool and an elevated fecal pH indicates a reduction of these species in the gut, which has been reported in many studies (19, 20, 29).

Confidential: For Review Only

While *Clostridium* cluster I and cluster XI include pathogenic species *Clostridium perfrigens, Clostridium tetani*, and *Clostridium difficile,* most of the *Clostridia* maintain a commensal relationship with the host (20). Gut commensal *Clostridia* consist of gram-positive, rod-shaped bacteria and clusters XIVa and IV make up a substantial part (10-40%) of the total bacteria in the gut microbiota (20). These commensals perform their metabolic functions through the release of butyrate, which is an important SCFA, that lowers the pH in the colon and contributes in stool acidification (20). "*Bacillus bifidus*" or *Bifidobacterium* is a Gram-positive, acidiphilic bacteria (30) belonging to the *Bifidobacteriaceae* family, has the capability to consume human milk oligosachharides (HMOs) and convert them to acidic end products with a meaningful effect on fecal pH (19). So, fecal pH acts as a useful marker for assessing gut commensal *Clostridia* and *Bifidobacterium* status in non-diarrheal children. However, as HMOs are involved in the acidification process related to *Bifidobacterium*, the commensal *Clostridia* are likely to have an important role in children of the studied age-group. However, using the data from Bangladesh Demographic and Health Survey 2004, a study showed that the average duration of breastfeeding in Bangladeshi children is 31.9 months (31). So, children in this region are breastfed along with complimentary feeding even beyond 12 months of age, which supports the concept of presence of *Bifido* in the gut of children up to this age (31). Characterization of microbiome (SCFA producers) in the stool could not be investigated in the present study, which is the only way to clarify this topic. Further studies are warranted to make exploration in this regard.

In the developing world, most of the linear growth faltering occurs in the first two years of life. However, very few studies exist on gut microbiota of children belonging to this period of life when the maturation of microbiota is the most required (7). As already mentioned, gut microbiota performs a wide range of protective, structural, and metabolic functions of the gut, all of which are critical to optimal nutrient absorption (8, 15-17). And an optimal nutrient absorption as well as a well-developed immune system is the key to optimum growth.

Confidential: For Review Only

SCFAs, in particular butyrate, are calorie-rich nutrients and are the preferred energy source for colonocytes and also a potent regulator of host metabolism (18). Their chronic depletion results in undernutrition and stunting (18). Furthermore, butyrate is an effector of colonization resistance to facultative anaerobic enteropathogens and reduction in butyrate promotes over-representation of aerobic enteropathogenic taxa *Streptococcus, Neisseria, Staphylococcus, Haemophilus, Campylobacter,* and *Escherichia/Shigella* genera constituting a bona fide dysbiosis, resulting in increased frequency and severity of gastrointestinal diseases, again leading to undernutrition and stunting (18, 32). Commensal *Clostridia* belonging to clusters XIVa and IV release butyrate to perform their metabolic functions and also been reported to be strong inducers of colonic T regulatory cell (Treg) accumulation (33), contributing to immune system development in children and reduction of this species is considered as a signature of stunting (18). On the other hand, *Bifidobacterium* is called the keystone infant gut symbiont (19) and *Bifidobacterium longum* subsp. *infantis* (*B. infantis*) is unique among all gut microbes to have the capacity to consume the full range of HMOs (34). HMOs serve as nutrients for colon bacteria, further promotes growth of nonpathogenic, bifidogenic microflora and prevents pathogenic microorganisms, e.g., *Campylobacter jejuni*, *E. coli*, *Vibrio cholera*, *Shigella* and *Salmonella* strains from adhesion to the host's epithelial surface and protect against infections and diarrhea (35).

This discussion makes it evident that commensal *Clostridia* and *Bifidobacteria* play a significant role for optimum child growth, both by facilitating nutrient absorption and providing with immunity, and studies show that these species are abundant in the gut of children with optimum linear growth and markedly decreased in the gut of stunted children (4, 7). And as fecal pH is an indirect yet novel measuring tool of their abundance in the non-diarrheal infant gut, which in turn is strongly associated to linear growth, fecal pH might just have emerged as a determinant of linear growth in early childhood, as we have found in this study.

Confidential: For Review Only

Another significant finding of this study is the positive association of maternal height with linear growth of the children. However, this has been established by previous studies (13). Short maternal height results in low birth weight of the child (36) which in turn results in reduced length-for-age of their offspring, pointing to the importance of intergenerational effects (13). Nutritional improvement during adolescence, pregnancy and early childhood has to be properly ensured to overcome this effect (37).

Limitations

This study was conducted as a part of the Bangladesh Environmental Enteric Dysfunction (BEED) study and the target population of the parent study were only stunted (length-for-age Z-scores (LAZ) <-2) and at risk of stunting (LAZ <-1 to -2) children and therefore, by definition were not completely healthy. The pH results of healthy children could not be examined or compared. Characterization of microbiome (SCFA producers) and SCFA profiles in the stool of the children with and without low pH was also not investigated.

Conclusion

Fecal pH and maternal height were found to have significant association with linear growth in early childhood. Previous studies have already established the association between maternal height and linear growth of the child. In this study, fecal pH has emerged as a determinant of linear growth in early childhood.

Confidential: For Review Only

What is already known on this topic

- Chronic malnutrition, manifested as linear growth failure or stunting, is the most common form of malnutrition across the globe
- Alteration in gut microbiota has impact on linear growth and microbiota of children with optimum linear growth are enriched in commensal *Clostridia* and *Bifidobacteria*
- Abundance of commensal *Clostridia* and *Bifidobacteria* contribute to formation of acidic stool and reduction of these species lead to an elevation in fecal pH

What this study adds

- There is a significantly inverse association between fecal pH and linear growth, making fecal pH a possible determinant of linear growth
- A significantly positive association exist between maternal height and LAZ score of the child

Confidential: For Review Only

References

1. Black RE, Victora CG, Walker SP, Bhutta ZA, Christian P, De Onis M, et al. Maternal and child undernutrition and overweight in low-income and middle-income countries. The lancet. 2013;382(9890):427-51.
2. Farràs M, Chandwe K, Mayneris-Perxachs J, Amadi B, Louis-Auguste J, Besa E, et al. Characterizing the metabolic phenotype of intestinal villus blunting in Zambian children with severe acute malnutrition and persistent diarrhea. PloS one. 2018;13(3):e0192092.
3. Amadi B, Besa E, Zyambo K, Kaonga P, Louis-Auguste J, Chandwe K, et al. Impaired barrier function and autoantibody generation in malnutrition enteropathy in Zambia. EBioMedicine. 2017;22:191-9.
4. Dinh DM, Ramadass B, Kattula D, Sarkar R, Braunstein P, Tai A, et al. Longitudinal analysis of the intestinal microbiota in persistently stunted young children in South India. PLoS One. 2016;11(5):e0155405.
5. Grantham-McGregor S, Cheung YB, Cueto S, Glewwe P, Richter L, Strupp B, et al. Developmental potential in the first 5 years for children in developing countries. The lancet. 2007;369(9555):60-70.
6. Harper KM, Mutasa M, Prendergast AJ, Humphrey J, Manges AR. Environmental enteric dysfunction pathways and child stunting: A systematic review. PLoS neglected tropical diseases. 2018;12(1):e0006205.
7. Kane AV, Dinh DM, Ward HD. Childhood malnutrition and the intestinal microbiome. Pediatric research. 2015;77(1-2):256.
8. Kau AL, Ahern PP, Griffin NW, Goodman AL, Gordon JI. Human nutrition, the gut microbiome and the immune system. Nature. 2011;474(7351):327.
9. Gordon JI, Dewey KG, Mills DA, Medzhitov RM. The human gut microbiota and undernutrition. Science translational medicine. 2012;4(137):137ps12-ps12.
10. Dewey KG, Begum K. Long-term consequences of stunting in early life. Maternal & child nutrition. 2011;7:5-18.
11. Keusch GT, Rosenberg IH, Denno DM, Duggan C, Guerrant RL, Lavery JV, et al. Implications of acquired environmental enteric dysfunction for growth and stunting in infants and children living in low-and middle-income countries. Food and nutrition bulletin. 2013;34(3):357-64.
12. Keusch GT, Denno DM, Black RE, Duggan C, Guerrant RL, Lavery JV, et al. Environmental enteric dysfunction: pathogenesis, diagnosis, and clinical consequences. Clinical Infectious Diseases. 2014;59(suppl_4):S207-S12.
13. Investigators M-EN. Childhood stunting in relation to the pre-and postnatal environment during the first 2 years of life: The MAL-ED longitudinal birth cohort study. PLoS medicine. 2017;14(10):e1002408.
14. Gough EK, Stephens DA, Moodie EE, Prendergast AJ, Stoltzfus RJ, Humphrey JH, et al. Linear growth faltering in infants is associated with Acidaminococcus sp. and community-level changes in the gut microbiota. Microbiome. 2015;3(1):24.
15. Bäckhed F, Ley RE, Sonnenburg JL, Peterson DA, Gordon JI. Host-bacterial mutualism in the human intestine. science. 2005;307(5717):1915-20.
16. O'Hara AM, Shanahan F. The gut flora as a forgotten organ. EMBO reports. 2006;7(7):688-93.
17. Evans JM, Morris LS, Marchesi JR. The gut microbiome: the role of a virtual organ in the endocrinology of the host. J Endocrinol. 2013;218(3):R37-47.
18. Vonaesch P, Morien E, Andrianonimiadana L, Sanke H, Mbecko J-R, Huus KE, et al. Stunted childhood growth is associated with decompartmentalization of the gastrointestinal tract and overgrowth of oropharyngeal taxa. Proceedings of the National Academy of Sciences. 2018;115(36):E8489-E98.

Confidential: For Review Only

19. Henrick BM, Hutton AA, Palumbo MC, Casaburi G, Mitchell RD, Underwood MA, et al. Elevated fecal pH indicates a profound change in the breastfed infant gut microbiome due to reduction of Bifidobacterium over the past century. mSphere. 2018;3(2):e00041-18.
20. Lopetuso LR, Scaldaferri F, Petito V, Gasbarrini A. Commensal Clostridia: leading players in the maintenance of gut homeostasis. Gut pathogens. 2013;5(1):23.
21. Mahfuz M, Das S, Mazumder RN, Rahman MM, Haque R, Bhuiyan MMR, et al. Bangladesh Environmental Enteric Dysfunction (BEED) study: protocol for a community-based intervention study to validate non-invasive biomarkers of environmental enteric dysfunction. BMJ open. 2017;7(8):e017768.
22. Osuka A, Shimizu K, Ogura H, Tasaki O, Hamasaki T, Asahara T, et al. Prognostic impact of fecal pH in critically ill patients. Critical Care. 2012;16(4):R119.
23. Gasbarrini A, Corazza G, Gasbarrini G, Montalto M, Di MS, Basilisco G, et al. Methodology and indications of H2-breath testing in gastrointestinal diseases: the Rome Consensus Conference. Alimentary pharmacology & therapeutics. 2009;29:1-49.
24. Stein AD, Wang M, Martorell R, Norris SA, Adair LS, Bas I, et al. Growth patterns in early childhood and final attained stature: data from five birth cohorts from low-and middle-income countries. American Journal of Human Biology. 2010;22(3):353-9.
25. Psaki SR, Seidman JC, Miller M, Gottlieb M, Bhutta ZA, Ahmed T, et al. Measuring socioeconomic status in multicountry studies: results from the eight-country MAL-ED study. Population health metrics. 2014;12(1):8.
26. Kvissberg MA, Dalvi PS, Kerac M, Voskuijl W, Berkley JA, Priebe MG, et al. Carbohydrate malabsorption in acutely malnourished children and infants: a systematic review. Nutrition reviews. 2015;74(1):48-58.
27. Betrán AP, Ye J, Moller A-B, Zhang J, Gülmezoglu AM, Torloni MR. The increasing trend in caesarean section rates: global, regional and national estimates: 1990-2014. PloS one. 2016;11(2):e0148343.
28. Duranti S, Lugli GA, Mancabelli L, Turroni F, Milani C, Mangifesta M, et al. Prevalence of antibiotic resistance genes among human gut-derived bifidobacteria. Appl Environ Microbiol. 2017;83(3):e02894-16.
29. Rivera-Chávez F, Zhang LF, Faber F, Lopez CA, Byndloss MX, Olsan EE, et al. Depletion of butyrate-producing Clostridia from the gut microbiota drives an aerobic luminal expansion of Salmonella. Cell host & microbe. 2016;19(4):443-54.
30. Logan W. The intestinal flora of infants and young children. The Journal of Pathology and Bacteriology. 1913;18(1):527-51.
31. Akter S, Rahman MM. Duration of breastfeeding and its correlates in Bangladesh. Journal of health, population, and nutrition. 2010;28(6):595.
32. Vonaesch P, Anderson M, Sansonetti PJ. Pathogens, microbiome and the host: emergence of the ecological Koch's postulates. FEMS microbiology reviews. 2018;42(3):273-92.
33. Atarashi K, Tanoue T, Shima T, Imaoka A, Kuwahara T, Momose Y, et al. Induction of colonic regulatory T cells by indigenous Clostridium species. Science. 2011;331(6015):337-41.
34. Underwood MA, German JB, Lebrilla CB, Mills DA. Bifidobacterium longum subspecies infantis: champion colonizer of the infant gut. Pediatric research. 2015;77(1-2):229.
35. Bode L. Recent advances on structure, metabolism, and function of human milk oligosaccharides. The Journal of Nutrition. 2006;136(8):2127-30.
36. Han Z, Lutsiv O, Mulla S, McDonald SD, Group KS. Maternal height and the risk of preterm birth and low birth weight: a systematic review and meta-analyses. Journal of Obstetrics and Gynaecology Canada. 2012;34(8):721-46.
37. Garza C, Borghi E, Onyango AW, de Onis M, Group WMGRS. Parental height and child growth from birth to 2 years in the WHO M ulticentre G rowth R eference S tudy. Maternal & child nutrition. 2013;9:58-68.

Confidential: For Review Only

Table Legends

Table 1. Demographic characteristics and results of the respondents

**Characteristics**	**Mean ± SD* (n=100)**
**Mean age of children (Months)**	15.16 ± 2.15
**Female,%**	57%
**Mother's age (Years)**	25.2 ± 6.3
**Mother's schooling years**	5.96 ± 2.8
**Mother's currently working status**	
Yes	25%
No	75%
**Father's occupation**	
Laborer	19%
Service holder/ Businessman	22%
Driver	28%
Factory worker	17%
Others (unemployment, retired)	14%
**Mother's hand wash practice**	
**Wash hand with soap after helping child defecate,%**	
Always	75%
Sometimes	22%
Rarely	3%
Never	0%
**Wash hand with soap before preparing food,%**	
Always	15%
Sometimes	36%
Rarely	11%
Never	38%
**Monthly family income in USD*, median (IQR*)**	178 (119, 237)

* SD= Standard deviation; USD= United States Dollars; IQR=Inter-quartile range

Confidential: For Review Only

Table 2. Association of LAZ scores with fecal pH

Variables	Unadjusted			Adjusted		
	B*	Standard Error	Sig.*	B*	Standard Error	Sig.*
Age (in months)	0.02	0.04	0.56	0.02	0.03	0.57
Sex	0.17	0.16	0.29	0.15	0.14	0.29
Mothers height (in cm)	0.04	0.02	0.02	0.04	0.01	**0.01**
Fever (in last 14 days)	0.05	0.58	0.93			
Antibiotics taken (in last 14 days)	0.56	0.81	0.49			
Fecal pH	-0.34	0.07	0.00	-0.34	0.06	**0.00**
SIBO*	0.21	0.24	0.39			
MDD*	0.05	0.17	0.76	0.13	0.15	0.40
Food Group 1 (Grain, roots & tubers)	-0.18	0.81	0.83			
Food Group 2 (Legumes & nuts)	-0.28	0.16	0.09			
Food Group 3 (Milk, yogurt & cheese)	0.06	0.16	0.72			
Food Group 4 (Meat, fish & poultry)	0.05	0.16	0.77			
Food Group 5 (Eggs)	0.00	0.16	0.98			
Food Group 6 (Vitamin-A rich fruits & vegetables)	-0.32	0.18	0.07			
Food Group 7 (Other fruits & vegetables)	0.17	0.16	0.29			
Currently Breastfeeding	0.05	0.21	0.82			
WAMI*	1.06	0.63	0.09	0.85	0.56	0.13

*B=Regression co-efficient; Sig= Significance level; SIBO=Small intestinal bacterial overgrowth; MDD=Minimum dietary diversity; WAMI= incorporation of several aspects of socio-economic status: access to improved **W**ater and sanitation, the selected approach to measuring household wealth (**A**ssets), **M**aternal education, and **I**ncome

Confidential: For Review Only

Figure Legend

Figure 1. Scatter diagram showing negative correlation between fecal pH and LAZ scores

Confidential: For Review Only

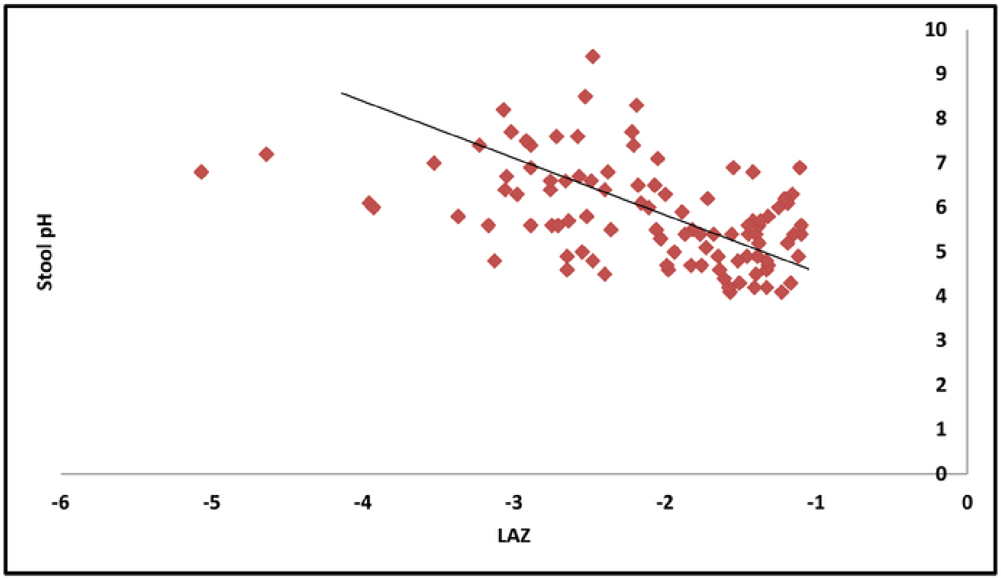

58x33mm (300 x 300 DPI)

BMJ Paediatrics Open

Association of fecal pH with childhood stunting: Results from a cross-sectional study

Journal:	*BMJ Paediatrics Open*
Manuscript ID	bmjpo-2019-000549.R1
Article Type:	Original article
Date Submitted by the Author:	19-Aug-2019
Complete List of Authors:	Hossain, Md. Shabab; International Centre for Diarrhoeal Disease Research Bangladesh,<br>Das, Subhasish; International Centre for Diarrhoeal Disease Research Bangladesh<br>Gazi, Md. Amran; International Centre for Diarrhoeal Disease Research Bangladesh<br>Alam, Md. Ashraful ; International Centre for Diarrhoeal Disease Research Bangladesh<br>Haque, Nur Muhammad Shahedul ; International Centre for Diarrhoeal Disease Research Bangladesh<br>Mahfuz, Mustafa; International Centre for Diarrhoeal Disease Research Bangladesh<br>Ahmed, Tahmeed; International Centre for Diarrhoeal Disease Research Bangladesh, Nutrition and Clinical Services Division (NCSD)<br>Damman, Chris J. ; University of Washington; Bill and Melinda Gates Foundation
Keywords:	Growth

SCHOLARONE™
Manuscripts

Association of fecal pH with childhood stunting: Results from a cross-sectional study

Md. Shabab Hossain[1], Subhasish Das[1], Md. Amran Gazi[1], Md. Ashraful Alam[1], Nur Muhammad Shahedul Haque[1], Mustafa Mahfuz[1], Tahmeed Ahmed[1], Chris J. Damman[2,3]

[1]Nutrition and Clinical Services Division, International Centre for Diarrheal Disease Research, Bangladesh (icddr,b), Dhaka, Bangladesh

[2]Program Officer, Bill and Melinda Gates Foundation, Seattle, Washington, USA

[3]Division of Gastroenterology, Department of Medicine, University of Washington, Seattle, Washington, USA

Name and email of all authors:

Md. Shabab Hossain: dr.shabab@icddrb.org

Subhasish Das: subhasish.das@icddrb.org

Md. Amran Gazi: amran.gazi@icddrb.org

Md. Ashraful Alam: mashraful@icddrb.org

Nur Muhammad Shahedul Haque: shahedhaq@icddrb.org

Mustafa Mahfuz: mustafa@icddrb.org

Tahmeed Ahmed: tahmeed@icddrb.org

Chris J. Damman: Chris.Damman@gatesfoundation.org

Address for correspondence:

Tahmeed Ahmed

Nutrition and Clinical Services Division, International Centre for Diarrhoeal Disease Research, Bangladesh (icddr,b), 68 Shaheed Tajuddin Ahmed Sarani, Mahakhali, Dhaka, Bangladesh

Phone: +880 1713044799; Email: tahmeed@icddrb.org

Confidential: For Review Only

Key words: Fecal pH; growth; children; Bangladesh

Manuscript Word count: 2485

Number of figures: 01

Number of tables: 02

Source of Funding

This protocol is supported by the Bill and Melinda Gates Foundation under its Global Health Program. Project investment ID is OPP1136751.

(https://www.gatesfoundation.org/How-We-Work/Quick-Links/Grants-Database/Grants/2015/11/OPP1136751)

Trial registration number

ClinicalTrials.gov ID: NCT02812615

(https://clinicaltrials.gov/ct2/results?cond=NCT02812615&term=&cntry=&state=&city=&dist=)

Conflicts of Interest

None declared

Acknowledgements

This research protocol was funded by *the Bill and Melinda Gates Foundation (BMGF) under its Global Health Program. Project investment ID is OPP1136751*. icddr,b acknowledges with gratitude the commitment of the BMGF to its research efforts. icddr,b is also grateful to the Governments of Bangladesh, Canada, Sweden and the UK for providing core/unrestricted support. We express our sincere thanks to our colleagues at the International Centre for Diarrhoeal Disease Research, Bangladesh and the study participants.

Confidential: For Review Only

Author contributions

CJD and TA originated the idea for the study and TA led the protocol design. MSH, SD and TA participated in the design of the study. MSH, SD, MAG, NMSH, MM and TA conducted the study and supervised the sample and data collection. MSH and MAG performed and supervised the sample analysis. MSH, SD, MAA, MM and TA were involved in data analysis. MSH, SD and TA interpreted the results. MSH, SD, MM and TA were involved in manuscript writing. All authors read and approved the final manuscript.

Confidential: For Review Only

Abstract

Background: Gut microbiota plays an important role in growth of children. The gut of children with optimum growth is enriched in certain species, especially *Bifidobacteria* and *Clostridia*. *Bifidobacteria* and commensal *Clostridia* both contribute to formation of acidic stool, and an elevated fecal pH indicates reduction of these species in the gut. The purpose of the study was to investigate the association of fecal pH with childhood stunting.

Methods: In this cross-sectional study, one hundred children with length-for-age Z score (LAZ) <-1 aged between 12-18 months were enrolled from the ongoing Bangladesh Environmental Enteric Dysfunction (BEED) study conducted in Dhaka, Bangladesh. LAZ was measured by anthropometry and data on factors affecting linear growth was recorded. Fecal pH measurement was done using pH meter on freshly collected non-diarrheal fecal samples following standard procedure. Multiple quantile regression was done to quantify the relation between fecal pH and LAZ scores.

Results: The mean LAZ and fecal pH of the children were -2.12±0.80 and 5.84±1.11 respectively. Pearson correlation analysis showed a statistically significant negative correlation between stool pH and the LAZ scores (p<0.01). After inclusion of other factors affecting linear growth into the regression model, a statistically significant inverse association was observed between fecal pH and LAZ score (p<0.01).

Conclusion: Elevated fecal pH was found to have significant association with stunted growth. As an indicator of gut microbiota status, fecal pH might have emerged as a possible indirect determinant of childhood stunting.

Confidential: For Review Only

Introduction

Malnutrition is the underlying cause of almost half of all deaths in under five children, estimated to be more than 3 million child deaths annually (1). The burden of malnutrition is very high in children living in low-and-middle-income countries (LMICs) (1). Chronic malnutrition, which is manifested as linear growth failure or stunting (2), is the most common form of malnutrition across the globe, affecting about one-third of children in developing countries (3). Among these developing countries, countries from the southern Asia, specifically Bangladesh, India, Pakistan, Nepal and Bhutan harbors a substantially high number of stunted children (4). Stunted children suffer from reduced neuro-developmental potential and poor cognitive function (2), thus fail to reach their full developmental potential (5), leading to sub-standard educational performance and economic productivity in the later phases of life (6). The etiology of stunting is poorly understood (4) and though poverty-associated food insecurity might play a vital role, the etiology of this condition is far more complex (7). Several factors including intrauterine growth retardation, mother's height, impaired immunity and recurrent infections, small intestinal bacterial overgrowth (SIBO), environmental enteric dysfunction (EED), neuro-endocrine and hormonal factors, and host genetic factors compounded by food insecurity are all part of this vicious process (8-14). Recent studies suggest that alteration in the gut microbiota is also a part of this process (4, 7-9, 15).

A healthy gut microbiota is essential to human health (7). It performs diverse protective, structural, and metabolic functions of the gut; for instance, production of short-chain fatty acids (SCFA), which act as the primary nutrient source for the colonic epithelium, intestinal epithelial cell proliferation and development of the mucosal immune system, production of certain vitamins, promoting mineral absorption, influencing metabolic rates and appetite, induction of host genes for nutrient uptake; all of which are critical for optimal nutrient absorption (8, 16-18). On the other hand, dysbiosis refers to an altered microbiota composition that could lead to a number of disease states or malnutrition even in the face of adequate food intake (7).

Confidential: For Review Only

Despite the fact that chronic malnutrition is more prevalent than acute malnutrition globally, most published studies have concentrated on the role of gut microbiota in acute malnutrition. The limited studies that exist show that, gut microbiota of stunted children are predominant in bacteria belonging to the inflammogenic taxa including those in the Desulfovibrio genus and Campylobacterales order (4). On the contrary, the gut microbiota of children with optimum growth are enriched in certain species, especially *Bifidobacteria* (4, 7), which is referred to as the keystone infant gut symbiont (19), and the commensal *Clostridia* (20).. Fecal pH, a probable determinant whose relationship with growth has never been explored to the best of our knowledge, is strongly driven by the abundance of *Bifidobacteria* and the butyrate producing commensal *Clostridia*, especially clusters XIVa and IV, in the gut (19, 21). These commensals lower the pH in the colon contributing to formation of a more acidic stool and the reduction or loss of these species lead to an elevation in fecal pH (19).

Therefore, elevated fecal pH indicates a marked change in the infant gut microbiome, specifically a profound reduction of the mentioned species. Since fecal pH is an indirect yet novel tool for assessing *Bifidobacteria* and commensal *Clostridia* abundance in the infant gut, while their abundance share a significant association with childgrowth, we made an attempt to determine the association between child growth and fecal pH and to investigate if elevated fecal pH is associated with stunted growth in early childhood.

Methods

Study site and Data collection

This cross-sectional study was a part of the ongoing Bangladesh Environmental Enteric Dysfunction (BEED) study which is being conducted among the residents of *Bauniabadh* slum in Mirpur, Dhaka. BEED study is a community-based nutrition intervention study aimed to validate non-invasive biomarkers of environmental enteric dysfunction (EED) and better understand the disease pathogenesis. The details and overall design of the BEED study have already been published elsewhere (22). A written

Confidential: For Review Only

informed consent was obtained from the parents of children after explaining the aims and procedures of the study. For this particular study, a total of 100 children aged between 12-18 months with length-for-age Z-scores (LAZ) <-1 were enrolled from the same community from September 2018 to February 2019. Weight and length of the child along with mother's height was measured using standard protocol (23). Data regarding socio-economic, morbidity, dietary intake and breastfeeding was collected using hard copy questionnaires at the time of enrolment and prior to sample collection (24, 25). Dietary intake was assessed using qualitative food frequency questionnaires (FFQ) in which individual food items that was fed to each child during the last 24 hours were recorded (24, 25). This research was done without patient involvement. Patients were not invited to comment on the study design and were not consulted to develop patient relevant outcomes or interpret the results. Patients were not invited to contribute to the writing or editing of this document for readability or accuracy. Ethical approval was taken from Ethical Review Committee (ERC) of the Institutional Review Board (IRB) of icddr,b.

Measuring fecal pH

Prior to the nutritional intervention, the health workers waited for the children to defecate and fecal samples from the first bowel movement after enrolment were collected. Following standard operating procedures (19, 22, 26), fresh non-diarrheal fecal samples were collected immediately after defecation into a plastic pot and 1-2 grams from that stool sample was relocated in a sterile specimen container. 10 ml of de-ionized water was mixed and homogenized with this stool properly. The probes of the pH meter were completely dipped into this mixture and kept for one minute and the reading was recorded. The procedure was done within the first 2 hours of sample collection. The test was carried out using portable stool pH meter (Hanna Instruments, USA).

Small intestinal bacterial overgrowth (SIBO)

Presence of small intestinal bacterial overgrowth was determined by hydrogen breath test from breath samples taken from the children using BreathTracker SC (QuinTron Instrument Company, Milwaukee,

Confidential: For Review Only

USA). A total of ten breath samples were collected per case including a 3-hr fasting baseline sample followed by nine more samples at 20 minutes intervals after ingestion of glucose at 1 gram/kg body weight diluted in 5 ml/kg of water. A single difference of ≥ 12 ppm of hydrogen value from fasting baseline was considered to be SIBO positive (27).

Statistical analyses

Statistical analyses were performed using Stata version 13.0. Mean values and standard deviation (SD) of means were used to describe the distribution. Correlation between stool pH and LAZ scores was done by simple correlation analysis using Pearson correlation and visualized by scatter plots. As LAZ is the outcome variable, which was not normally distributed based on Shapiro-Wilk test, at first simple quantile regression was performed between LAZ scores with each individual factor. Results with a significance level at or below 0.2 were included in the multiple quantile regression model. In addition, age and sex were included in the final model due to the inconsistent growth pattern in children of either sex in this age group (28) and minimum dietary diversity (MDD) was included in the final model instead of the individual food groups. Multiple quantile regression was done to quantify the relation between fecal pH and LAZ scores after adjusting for age, sex, maternal height, MDD, breastfeeding, morbidity, antibiotics taken in the last 14 days, SIBO and incorporation of several aspects of socio-economic status: access to improved **W**ater and sanitation, the selected approach to measuring household wealth (**A**ssets), **M**aternal education, and **I**ncome (i.e. the WAMI index) (29).

Results

A total of 100 children with mean age of 15±2 months underwent fecal pH examination. Among them, 57 (57%) were female and 43 (43%) were male. Table 1 shows the socio-demographic characteristics of the study population.

Confidential: For Review Only

The mean LAZ and fecal pH of the children were -2.12±0.80 and 5.84±1.11 respectively. Pearson correlation between fecal pH and LAZ scores showed a statistically significant negative correlation (-0.46, $p<0.01$). Scatter plot (Figure-1) showed a similar correlation between stool pH and the LAZ scores as well.

Results of the simple quantile regression with a significance level at or below 0.2 and in addition age, sex and MDD were included in the multiple quantile regression model. SIBO was not significantly correlated with stunting in the simple quantile regression (p=0.14) and thus was not included in the multiple model. A statistically significant inverse association was observed between fecal pH with LAZ score of the children (Table 2). A single unit increase in fecal pH was associated with 0.32 unit decrease in the LAZ score of the child.

Discussion

Our study revealed that, elevated fecal pH has a significant association with stunted growth in early childhood. Though studies have shown significant association of fecal pH with severe acute malnutrition (SAM) (30), a thorough literature review yielded no results on the relationship between fecal pH with chronic malnutrition or stunted growth.

A trend of increasing infant fecal pH in children is observed over the past century which demonstrated a change in fecal pH from 5.0 to 6.5 from 1926 to 2017 (19). This elevation of fecal pH is consistent with reported reduction of certain important gut microflora. Factors responsible for reduction of these important microbiota from the infant gut over the past century comprises of an increase in consumption of human milk replacers (e.g., infant formula) which lack the bacterial selectivity of human milk (19), increased caesarean section delivery which limits the natural fecal-oral transfer from mother to infant associated with vaginal delivery (31) and overuse of antibiotics which alter the acquisition of gut microbes by the infant (32). *Bifidobacteria* and butyrate producing commensal *Clostridia* both contribute

Confidential: For Review Only

to formation of acidic stool and an elevated fecal pH indicates a reduction of these species in the gut, which has been reported in many studies (19, 21, 33).

While *Clostridium* cluster I and cluster XI include pathogenic species *Clostridium perfrigens, Clostridium tetani*, and *Clostridium difficile,* most of the *Clostridia* maintain a commensal relationship with the host (21). Gut commensal *Clostridia* consist of gram-positive, rod-shaped bacteria and clusters XIVa and IV make up a substantial part (10-40%) of the total bacteria in the gut microbiota (21). These commensals perform their metabolic functions through the release of butyrate, which is an important SCFA, that lowers the pH in the colon and contributes in stool acidification (21). "*Bacillus bifidus*" or *Bifidobacterium* is a Gram-positive, acidiphilic bacteria (34) belonging to the *Bifidobacteriaceae* family, has the capability to consume human milk oligosachharides (HMOs) and convert them to acidic end products with a meaningful effect on fecal pH (19). So, fecal pH acts as a useful marker for assessing gut *Bifidobacterium* and commensal *Clostridia* status in non-diarrheal children. However, as HMOs are involved in the acidification process related to *Bifidobacterium*, the commensal *Clostridia* are likely to have an important role in children of the studied age-group. However, using the data from Bangladesh Demographic and Health Survey 2004, a study showed that the average duration of breastfeeding in Bangladeshi children is 31.9 months (35). So, children in this region are usually breastfed along with complimentary feeding beyond 24 months of age, which supports the concept of presence of *Bifido* in the gut of children up to this age (35). Characterization of microbiome (SCFA producers) in the stool could not be investigated in the present study, which is the only way to clarify this topic. Further studies are warranted to make exploration in this regard.

In the developing world, most of the linear growth faltering occurs in the first two years of life. However, very few studies exist on gut microbiota of children belonging to this period of life when the maturation of microbiota is the most required (7). As already mentioned, gut microbiota performs a wide range of protective, structural, and metabolic functions of the gut, all of which are critical to optimal nutrient

Confidential: For Review Only

absorption (8, 16-18). And an optimal nutrient absorption as well as a well-developed immune system is the key to optimum growth. A recent study done in Guatemala has shown that both *Bifido* and *Clostridia* have biologically plausible effect on childhood stunting, however, the former was found to be more evident than the latter (36). SIBO was not significantly correlated with stunting in the present study. This might be due to the fact that all the participants here were non-diarrheal, while few studies indicate that SIBO is associated with chronic diarrhea (37, 38).

SCFAs, in particular butyrate, are calorie-rich nutrients and are the preferred energy source for colonocytes and also a potent regulator of host metabolism (20). Their chronic depletion results in undernutrition and stunting (20). Furthermore, butyrate is an effector of colonization resistance to facultative anaerobic enteropathogens and reduction in butyrate promotes over-representation of aerobic enteropathogenic taxa *Streptococcus, Neisseria, Staphylococcus, Haemophilus, Campylobacter,* and *Escherichia/Shigella* genera constituting a bona fide dysbiosis, resulting in increased frequency and severity of gastrointestinal diseases, again leading to undernutrition and stunting (20, 39). Commensal *Clostridia* belonging to clusters XIVa and IV release butyrate to perform their metabolic functions and also been reported to be strong inducers of colonic T regulatory cell (Treg) accumulation (40), contributing to immune system development in children and reduction of this species is considered as a signature of stunting (20). On the other hand, *Bifidobacterium* is called the keystone infant gut symbiont (19) and *Bifidobacterium longum* subsp. *infantis* (*B. infantis*) is unique among all gut microbes to have the capacity to consume the full range of HMOs (41). HMOs serve as nutrients for colon bacteria, further promotes growth of nonpathogenic, bifidogenic microflora and prevents pathogenic microorganisms, e.g., *Campylobacter jejuni*, *E. coli*, *Vibrio cholera*, *Shigella* and *Salmonella* strains from adhesion to the host's epithelial surface and protect against infections and diarrhea (42).

This discussion makes it evident that *Bifidobacteria* and commensal *Clostridia* play a significant role for optimum child growth, both by facilitating nutrient absorption and providing with immunity, and studies

Confidential: For Review Only

show that these species are abundant in the gut of children with optimum growth and markedly decreased in the gut of stunted children (4, 7). And as fecal pH is an indirect yet novel measuring tool of their abundance in the non-diarrheal infant gut, which in turn is strongly associated withgrowth, elevated fecal pH might just have emerged as a possible indirect determinant of stunted growth in early childhood, as we have found in this study.

Limitations

This study was conducted as a part of the Bangladesh Environmental Enteric Dysfunction (BEED) study and the target population of the parent study were only stunted (length-for-age Z-scores (LAZ) <-2) and at risk of stunting (LAZ <-1 to -2) children and therefore, by definition were not completely healthy. The pH results of healthy children could not be examined or compared. Characterization of microbiome (SCFA producers) and SCFA profiles in the stool of the children with and without low pH was also not investigated.

Conclusion

Elevated fecal pH was found to have significant association with stunted growth in early childhood. In this study, as an indicator of gut microbiota status, fecal pH came out as a possible indirect determinant of childhood stunting. Characterization of microbiome (SCFA producers) in the stool could not be investigated in the present study, which is the only way to clarify this topic. Further studies are warranted to make exploration in this regard.

Confidential: For Review Only

What is already known on this topic

- Chronic malnutrition, manifested as linear growth failure or stunting, is the most common form of malnutrition across the globe
- Alteration in gut microbiota has impact on stunted growth and microbiota of children with optimum growth are enriched in commensal *Clostridia* and *Bifidobacteria*
- Abundance of commensal *Clostridia* and *Bifidobacteria* contribute to formation of acidic stool and reduction of these species lead to an elevation in fecal pH

What this study adds

- There is a significantly inverse association between fecal pH and child growth, making elevated fecal pH a possible indirect determinant of childhood stunting

Confidential: For Review Only

References

1. Black RE, Victora CG, Walker SP, Bhutta ZA, Christian P, De Onis M, et al. Maternal and child undernutrition and overweight in low-income and middle-income countries. The lancet. 2013;382(9890):427-51.
2. Farràs M, Chandwe K, Mayneris-Perxachs J, Amadi B, Louis-Auguste J, Besa E, et al. Characterizing the metabolic phenotype of intestinal villus blunting in Zambian children with severe acute malnutrition and persistent diarrhea. PloS one. 2018;13(3):e0192092.
3. Amadi B, Besa E, Zyambo K, Kaonga P, Louis-Auguste J, Chandwe K, et al. Impaired barrier function and autoantibody generation in malnutrition enteropathy in Zambia. EBioMedicine. 2017;22:191-9.
4. Dinh DM, Ramadass B, Kattula D, Sarkar R, Braunstein P, Tai A, et al. Longitudinal analysis of the intestinal microbiota in persistently stunted young children in South India. PLoS One. 2016;11(5):e0155405.
5. Grantham-McGregor S, Cheung YB, Cueto S, Glewwe P, Richter L, Strupp B, et al. Developmental potential in the first 5 years for children in developing countries. The lancet. 2007;369(9555):60-70.
6. Harper KM, Mutasa M, Prendergast AJ, Humphrey J, Manges AR. Environmental enteric dysfunction pathways and child stunting: A systematic review. PLoS neglected tropical diseases. 2018;12(1):e0006205.
7. Kane AV, Dinh DM, Ward HD. Childhood malnutrition and the intestinal microbiome. Pediatric research. 2015;77(1-2):256.
8. Kau AL, Ahern PP, Griffin NW, Goodman AL, Gordon JI. Human nutrition, the gut microbiome and the immune system. Nature. 2011;474(7351):327.
9. Gordon JI, Dewey KG, Mills DA, Medzhitov RM. The human gut microbiota and undernutrition. Science translational medicine. 2012;4(137):137ps12-ps12.
10. Dewey KG, Begum K. Long-term consequences of stunting in early life. Maternal & child nutrition. 2011;7:5-18.
11. Keusch GT, Rosenberg IH, Denno DM, Duggan C, Guerrant RL, Lavery JV, et al. Implications of acquired environmental enteric dysfunction for growth and stunting in infants and children living in low- and middle-income countries. Food and nutrition bulletin. 2013;34(3):357-64.
12. Keusch GT, Denno DM, Black RE, Duggan C, Guerrant RL, Lavery JV, et al. Environmental enteric dysfunction: pathogenesis, diagnosis, and clinical consequences. Clinical Infectious Diseases. 2014;59(suppl_4):S207-S12.
13. Investigators M-EN. Childhood stunting in relation to the pre-and postnatal environment during the first 2 years of life: The MAL-ED longitudinal birth cohort study. PLoS medicine. 2017;14(10):e1002408.
14. Donowitz JR, Haque R, Kirkpatrick BD, Alam M, Lu M, Kabir M, et al. Small intestine bacterial overgrowth and environmental enteropathy in Bangladeshi children. MBio. 2016;7(1):e02102-15.
15. Gough EK, Stephens DA, Moodie EE, Prendergast AJ, Stoltzfus RJ, Humphrey JH, et al. Linear growth faltering in infants is associated with Acidaminococcus sp. and community-level changes in the gut microbiota. Microbiome. 2015;3(1):24.
16. Bäckhed F, Ley RE, Sonnenburg JL, Peterson DA, Gordon JI. Host-bacterial mutualism in the human intestine. science. 2005;307(5717):1915-20.
17. O'Hara AM, Shanahan F. The gut flora as a forgotten organ. EMBO reports. 2006;7(7):688-93.
18. Evans JM, Morris LS, Marchesi JR. The gut microbiome: the role of a virtual organ in the endocrinology of the host. J Endocrinol. 2013;218(3):R37-47.

Confidential: For Review Only

19. Henrick BM, Hutton AA, Palumbo MC, Casaburi G, Mitchell RD, Underwood MA, et al. Elevated fecal pH indicates a profound change in the breastfed infant gut microbiome due to reduction of Bifidobacterium over the past century. mSphere. 2018;3(2):e00041-18.
20. Vonaesch P, Morien E, Andrianonimiadana L, Sanke H, Mbecko J-R, Huus KE, et al. Stunted childhood growth is associated with decompartmentalization of the gastrointestinal tract and overgrowth of oropharyngeal taxa. Proceedings of the National Academy of Sciences. 2018;115(36):E8489-E98.
21. Lopetuso LR, Scaldaferri F, Petito V, Gasbarrini A. Commensal Clostridia: leading players in the maintenance of gut homeostasis. Gut pathogens. 2013;5(1):23.
22. Mahfuz M, Das S, Mazumder RN, Rahman MM, Haque R, Bhuiyan MMR, et al. Bangladesh Environmental Enteric Dysfunction (BEED) study: protocol for a community-based intervention study to validate non-invasive biomarkers of environmental enteric dysfunction. BMJ open. 2017;7(8):e017768.
23. Group WMGRS, de Onis M. Reliability of anthropometric measurements in the WHO Multicentre Growth Reference Study. Acta Paediatrica. 2006;95:38-46.
24. Kosek M, Guerrant RL, Kang G, Bhutta Z, Yori PP, Gratz J, et al. Assessment of environmental enteropathy in the MAL-ED cohort study: theoretical and analytic framework. Clinical Infectious Diseases. 2014;59(suppl_4):S239-S47.
25. The MAL-ED study: a multinational and multidisciplinary approach to understand the relationship between enteric pathogens, malnutrition, gut physiology, physical growth, cognitive development, and immune responses in infants and children up to 2 years of age in resource-poor environments. Clinical Infectious Diseases. 2014;59(suppl_4):S193-S206.
26. Osuka A, Shimizu K, Ogura H, Tasaki O, Hamasaki T, Asahara T, et al. Prognostic impact of fecal pH in critically ill patients. Critical Care. 2012;16(4):R119.
27. Gasbarrini A, Corazza G, Gasbarrini G, Montalto M, Di MS, Basilisco G, et al. Methodology and indications of H2-breath testing in gastrointestinal diseases: the Rome Consensus Conference. Alimentary pharmacology & therapeutics. 2009;29:1-49.
28. Stein AD, Wang M, Martorell R, Norris SA, Adair LS, Bas I, et al. Growth patterns in early childhood and final attained stature: data from five birth cohorts from low-and middle-income countries. American Journal of Human Biology. 2010;22(3):353-9.
29. Psaki SR, Seidman JC, Miller M, Gottlieb M, Bhutta ZA, Ahmed T, et al. Measuring socioeconomic status in multicountry studies: results from the eight-country MAL-ED study. Population health metrics. 2014;12(1):8.
30. Kvissberg MA, Dalvi PS, Kerac M, Voskuijl W, Berkley JA, Priebe MG, et al. Carbohydrate malabsorption in acutely malnourished children and infants: a systematic review. Nutrition reviews. 2015;74(1):48-58.
31. Betrán AP, Ye J, Moller A-B, Zhang J, Gülmezoglu AM, Torloni MR. The increasing trend in caesarean section rates: global, regional and national estimates: 1990-2014. PloS one. 2016;11(2):e0148343.
32. Duranti S, Lugli GA, Mancabelli L, Turroni F, Milani C, Mangifesta M, et al. Prevalence of antibiotic resistance genes among human gut-derived bifidobacteria. Appl Environ Microbiol. 2017;83(3):e02894-16.
33. Rivera-Chávez F, Zhang LF, Faber F, Lopez CA, Byndloss MX, Olsan EE, et al. Depletion of butyrate-producing Clostridia from the gut microbiota drives an aerobic luminal expansion of Salmonella. Cell host & microbe. 2016;19(4):443-54.
34. Logan W. The intestinal flora of infants and young children. The Journal of Pathology and Bacteriology. 1913;18(1):527-51.
35. Akter S, Rahman MM. Duration of breastfeeding and its correlates in Bangladesh. Journal of health, population, and nutrition. 2010;28(6):595.

Confidential: For Review Only

36. Voth-Gaeddert LE, Torres O, Maldonado J, Krajmalnik-Brown R, Rittmann BE, Oerther DB. Aflatoxin Exposure, Child Stunting, and Dysbiosis in the Intestinal Microbiome Among Children in Guatemala. Environmental Engineering Science. 2019.
37. Dukowicz AC, Lacy BE, Levine GM. Small intestinal bacterial overgrowth: a comprehensive review. Gastroenterology & hepatology. 2007;3(2):112.
38. Gasbarrini A, Lauritano EC, Gabrielli M, Scarpellini E, Lupascu A, Ojetti V, et al. Small intestinal bacterial overgrowth: diagnosis and treatment. Digestive diseases. 2007;25(3):237-40.
39. Vonaesch P, Anderson M, Sansonetti PJ. Pathogens, microbiome and the host: emergence of the ecological Koch's postulates. FEMS microbiology reviews. 2018;42(3):273-92.
40. Atarashi K, Tanoue T, Shima T, Imaoka A, Kuwahara T, Momose Y, et al. Induction of colonic regulatory T cells by indigenous Clostridium species. Science. 2011;331(6015):337-41.
41. Underwood MA, German JB, Lebrilla CB, Mills DA. Bifidobacterium longum subspecies infantis: champion colonizer of the infant gut. Pediatric research. 2015;77(1-2):229.
42. Bode L. Recent advances on structure, metabolism, and function of human milk oligosaccharides. The Journal of Nutrition. 2006;136(8):2127-30.

Confidential: For Review Only

Table Legends

Table 1. Demographic characteristics and results of the respondents

**Characteristics**	**Mean ± SD* (n=100)**
**Mean age of children (Months)**	15.16 ± 2.15
**Female,%**	57%
Fecal pH	5.84±1.11
Length for age Z-score (LAZ)	-2.12±0.80
Weight for age Z-score (WAZ)	-2.18±6.9
Weight for height z-score (WHZ)	-0.73±0.9
**Mother's age (Years)**	25.2 ± 6.3
**Mother's schooling years**	5.96 ± 2.8
**Mother's currently working status**	
Yes	25%
No	75%
**Father's occupation**	
Laborer	19%
Service holder/ Businessman	22%
Driver	28%
Factory worker	17%
Others (unemployment, retired)	14%
**Mother's hand wash practice**	
**Wash hand with soap after helping child defecate,%**	
Always	75%
Sometimes	22%
Rarely	3%
Never	0%
**Wash hand with soap before preparing food,%**	
Always	15%
Sometimes	36%
Rarely	11%
Never	38%
**Monthly family income in USD*, median (IQR*)**	178 (119, 237)

* SD= Standard deviation; USD= United States Dollars; IQR=Inter-quartile range

Confidential: For Review Only

Table 2. Association of LAZ scores with fecal pH

Variables	Unadjusted			Adjusted		
	β*	Standard Error	p-value *	β*	Standard Error	p-value *
Age (in months)	0.03	0.06	0.65	0.03	0.04	0.44
Sex	0.16	0.26	0.55	-0.02	0.18	0.93
Mothers height (in cm)	0.06	0.03	0.02	0.03	0.02	0.08
Fever (in last 14 days)	0.44	0.93	0.64			
Antibiotics taken (in last 14 days)	0.44	1.32	0.74			
Fecal pH	-0.36	0.07	<0.01	-0.32	0.08	**<0.01**
SIBO*	0.57	0.38	0.14			
MDD*	0.01	0.27	0.97	0.15	0.18	0.41
Food Group 1 (Grain, roots & tubers)	-0.06	1.32	0.96			
Food Group 2 (Legumes & nuts)	-0.35	0.24	0.15			
Food Group 3 (Milk, yogurt & cheese)	-0.13	0.26	0.62			
Food Group 4 (Meat, fish & poultry)	0.09	0.26	0.73			
Food Group 5 (Eggs)	-0.05	0.26	0.85			
Food Group 6 (Vitamin-A rich fruits & vegetables)	-0.66	0.25	0.09			
Food Group 7 (Other fruits & vegetables)	0.30	0.25	0.23			
Currently Breastfeeding	0.09	0.33	0.79			
WAMI*	0.29	1.02	0.78			

*β=Regression co-efficient; **p-value** = Significance level; SIBO=Small intestinal bacterial overgrowth; MDD=Minimum dietary diversity; WAMI= incorporation of several aspects of socio-economic status: access to improved **W**ater and sanitation, the selected approach to measuring household wealth (**A**ssets), **M**aternal education, and **I**ncome

Confidential: For Review Only

Figure Legend

Figure 1. Scatter diagram showing negative correlation between fecal pH and LAZ scores

Confidential: For Review Only

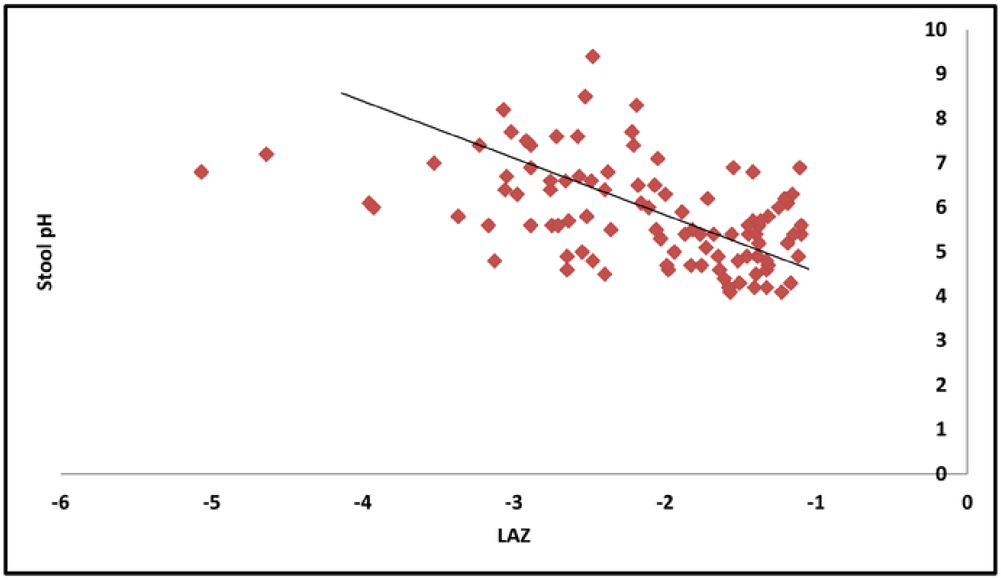

58x33mm (300 x 300 DPI)
